# Population differentiation and intraspecific genetic admixture in two *Eucryptorrhynchus* weevils (Coleoptera: Curculionidae) across northern China

**DOI:** 10.1002/ece3.8806

**Published:** 2022-04-06

**Authors:** Yu‐Jie Zhang, Wei Song, Li‐Jun Cao, Jin‐Cui Chen, Ary A. Hoffmann, Jun‐Bao Wen, Shu‐Jun Wei

**Affiliations:** ^1^ 12380 Beijing Key Laboratory for Forest Pests Control, College of Forestry Beijing Forestry University Beijing China; ^2^ 107624 Institute of Plant Protection Beijing Academy of Agriculture and Forestry Sciences Beijing China; ^3^ School of BioSciences Bio21 Institute The University of Melbourne Parkville Victoria Australia

**Keywords:** dispersal, *E. scrobiculatus*, *Eucryptorrhynchus brandti*, microsatellite, population genetics

## Abstract

Increasing damage of pests in agriculture and forestry can arise both as a consequence of changes in local species and through the introduction of alien species. In this study, we used population genetics approaches to examine population processes of two pests of the tree‐of‐heaven trunk weevil (TTW), *Eucryptorrhynchus brandti* (Harold) and the tree‐of‐heaven root weevil (TRW), *E*. *scrobiculatus* (Motschulsky) on the tree‐of‐heaven across their native range of China. We analyzed the population genetics of the two weevils based on ten highly polymorphic microsatellite markers. Population genetic diversity analysis showed strong population differentiation among populations of each species, with *F*_ST_ ranges from 0.0197 to 0.6650 and from −0.0724 to 0.6845, respectively. Populations from the same geographic areas can be divided into different genetic clusters, and the same genetic cluster contained populations from different geographic populations, pointing to dispersal of the weevils possibly being human‐mediated. Redundancy analysis showed that the independent effects of environment and geography could account for 93.94% and 29.70% of the explained genetic variance in TTW, and 41.90% and 55.73% of the explained genetic variance in TRW, respectively, indicating possible impacts of local climates on population genetic differentiation. Our study helps to uncover population genetic processes of these local pest species with relevance to control methods.

## Graphical abstract

Genetic structure of 13 and 11 populations of *Eucryptorrhynchus brandti* (TTW) and *E. scrobiculatus* (TRW) populations based on 10 microsatellite markers.

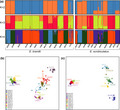

## 1 INTRODUCTION

In recent years, many new pests have damaged agricultural production and forestry operations (Riegler, 2018). These pests can be local species or introduced species from other distribution ranges. The local pests show increases in damage due to changes in the environment and management procedures or genetic adaption to local environments (Hoffmann & Sgro, 2011; Riegler, 2018); these include the direct effects of climate changes and management responses to them as well as the evolution of pesticide resistance (Bergé & Ricroch, 2010; Elbert et al., 2019). The spread of species is often facilitated by human activities (Bradshaw et al., 2016; Simberloff et al., 2013). In the past decade, human trade has increasingly promoted the movement of species beyond their historical distribution (Campagnaro et al., 2018; Gippet et al., 2019; Liebhold & Tobin, 2008). Identifying the possible sources of a new pest can provide essential information on developing control methods (Kirk et al., 2013a).

Population genetics approaches can help to reveal the population history of species and then infer whether the species is local or introduced outside of their native range (Antoine et al., 2017; Boissin et al., 2012b; Fraimout et al., 2017). For a pest species in its native range, genetic differentiation of populations is usually aligned to geographic distance (Wei et al., 2015), although the same pattern can be found in introduced species with stepping‐stone expansion (Cao et al., 2016; Lundhagen et al., 2013). For a newly introduced species, there is usually a lack of population differentiation or genetic isolation by geographic distance (Cao et al., 2017). The introduced populations usually share the same genetic cluster with their source populations (Beichman et al., 2018; Kirk et al., 2013b). Multiple introductions in different source populations can all lead to structured populations in the introduced area (Cao et al., 2017). Thus, based on patterns of population genetic differentiation and the connectivity among populations, it is possible to infer the population history of a species and provide insights into our understanding of newly outbreaking pests.

The tree‐of‐heaven trunk weevil (TTW), *Eucryptorrhynchus brandti* (Harold) and the tree‐of‐heaven root weevil (TRW), *E*. *scrobiculatus* (Motschulsky), are the only two wood‐boring species of *Eucryptorrhynchus* in China (Liu, 2016). The two species both only feed on the tree‐of‐heaven, *Ailanthus altissima* (Mill.) Swingle and its varieties *A*. *altissima* Qiantouchun in China and cause serious damage. They spend their larvae stage in the trunk (TTW) or root (TRW) of the host tree. There is a strict reproductive isolation between these two species of weevil. TRW lay eggs in the soil near the roots of *Ailanthus altissima*, while TTW lay eggs in the trunk of *Ailanthus altissima* (Zhang et al., 2019). In the past decades, TTW and TRW have caused increasing damage to *A*. *altissima* and become important pests in northern China (Wu et al., 2016) especially the Ningxia Hui Autonomous Region. This phenomenon is coincident with the widespread use of *A*. *altissima* as an ornamental tree planted along the sides of roads in northern China. In North America, TTW was used as a potential biological control agent to control *A*. *altissima* where it is an invasive plant (Herrick et al., 2012). Identifying the population evolutionary processes of these weevils may provide insights into local and more widespread movement of the two weevil pests, helping the development of control methods where these weevils are considered pests and ways of promoting their dispersal where the weevils are considered as biological control agents.

In this study, we examined the population genetic diversity and structure of the two weevils to infer their population history. Comparative study of the two weevils allows us to check if the two co‐occurring pests show similar population genetic processes. We hypothesized that the increasing damage by the two species in China is related to human‐mediated dispersal of seedings, based on the low flight ability of the two species and their life cycle. We also tested whether local climate can have an impact on the population genetic structure of the two species.

## 2 MATERIALS AND METHODS

### 2.1 Sample collection and genotyping

**FIGURE 1:**
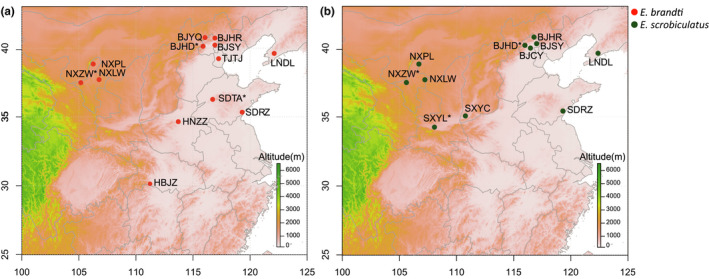
Collection sites for samples of *Eucryptorrhynchus brandti* (TTW, red) and *E*. *scrobiculatus* (TRW, green). Codes for collection sites are same as shown in Table 1. *Populations used for developing microsatellites of TTW and TRW

We collected 13 TTW and 11 TRW populations across their native range of China (Table 1, Figure 1). The two weevils are the primary pests of the tree‐of‐heaven, and we sampled sites separated by variable distances (30–2100 km). To avoid the collection of siblings, one adult individual was collected from each tree. The samples were kept in 100% ethanol and stored at −80°C before DNA extraction. Total genomic DNA was extracted from the legs of each adult using the DNeasy Blood & Tissue Kit (Qiagen, Hilden, Germany) according to the manufacturer's instructions.

**TABLE 1:** Collection information for specimens of *Eucryptorrhynchus brandti* (TTW) and *E*. *scrobiculatus* (TRW) used in the study

Code	Collection location	Longitude (E)	Latitude (N)	Collection date	No. (TTW/TRW)	Species
BJCY	Beijing, Chaoyang district	116.37	40.00	May−2018	0/5	TRW
BJHD	Beijing, Haidian district	116.22	40.04	July−2018	13/13	TTW and TRW
BJHR	Beijing, Huairou district	116.66	40.41	June−2018	12/13	TTW and TRW
BJSY	Beijing, Shunyi district	116.77	40.10	May−2020	20/10	TTW and TRW
BJYQ	Beijing, Yanqing district	115.89	40.51	June−2018	20/0	TTW
HBJZ	Hubei Province, Jingzhou	112.89	30.01	September−2020	5/0	TTW
HNZZ	Henan Province, Zhengzhou	113.56	34.67	June−2021	20/0	TTW
LNDL	Liaoning Province, Dalian	122.96	39.97	September−2019	10/7	TTW and TRW
NXLW	Ningxia Hui Autonomous Region, Lingwu	106.26	38.12	April−2018	12/12	TTW and TRW
NXPL	Ningxia Hui Autonomous Region, Pingluo	106.48	38.86	April−2018	13/13	TTW and TRW
NXZW	Ningxia Hui Autonomous Region, Zhongwei	105.12	37.50	April−2018	13/13	TTW and TRW
SDRZ	Shandong Province, Rizhao	118.95	35.66	July−2018	16/15	TTW and TRW
SDTA	Shandong Province, Taian	116.72	36.27	August−2018	12/0	TTW
SXYC	Shanxi Province, Yuncheng	111.48	35.29	July−2018	0/5	TRW
SXYL	Shanxi Province, Yangling	108.07	34.26	July−2018	0/12	TRW
TJTJ	Tianjin	117.20	39.13	September−2020	14/0	TTW

Ten microsatellite loci from Zhang et al. (2021) were used in our study (Table S1) and genotyped using the same methods. The size of amplified PCR products was determined using an ABI 3730xl DNA Analyzer with GeneScan 500 LIZ size standard. Alleles were identified with GENEMAPPER v 4.0 (Applied Biosystems, USA).

### 2.2 Population genetic diversity analysis

The number of alleles, observed heterozygosity (*H*_O_), and polymorphism information content (*PIC*) were analyzed by the macros in Microsatellite Tools (Park, 2001). The null allele frequencies were estimated using the software FreeNA (Chapuis & Estoup, 2007). Deviation from Hardy–Weinberg equilibrium (HWE) for each loci/population combination, linkage disequilibrium (LD) among loci within each population, pairwise mean population differentiation (*F*_ST_), and inbreeding coefficients (*F*_IS_) were estimated in GENEPOP v4.0.11 (Rousset, 2008). We used the HP‐RARE v1.1 (Kalinowski, 2005) to test allelic richness (*A*_R_) and allelic richness of private alleles (*P*_AR_) of each site. We used GENCLONE v2.0 (Sophie & Khalid, 2006) to estimate the total number of alleles (*A*_T_) and the unbiased expected heterozygosity (*H*_E_). We compared the number of alleles (*A*_S_) and heterozygosity (*H*_ES_) among samples with different sample sizes in GENCLONE.

### 2.3 Population genetic structure analysis

First, population genetic structure was investigated using STRUCTURE v2.3.4 (Earl & vonHoldt, 2012). We used 30 replicates of each *K* value from 1 to 10, with 200,000 Markov chain Monte Carlo iterations and a burn‐in of 100,000 iterations. The results were submitted to the online software Structure Harvester v0.6.94 (Earl & vonHoldt, 2012) to determine the optimal *K* value by a Delta *K* method. Membership coefficient matrices (Q‐matrices) associated with the optimal *K* were processed using CLUMPP v1.12 (Jakobsson & Rosenberg, 2007), and then visualized using DISTRUCT v1.1 (Taubert et al., 2019).

Second, discriminant analysis of principal component (DAPC) was used to analyze population genetic structure under default settings (Jombart et al., 2008), complementing the STRUCTURE analysis. This analysis was run using an R package *adegenet* v1.4‐2.

### 2.4 Gene flow analysis

We used the BAYESASS v3.0.4 (Wilson & Rannala, 2003) to estimate the recent migration rates among populations of the two species. First, we conducted preliminary runs (10,000,000 steps) to adjust mixing parameters for allele frequencies and inbreeding coefficients. Then, we carried on ten longer runs of 100,000,000 steps using different start seeds with a sampling frequency of every 1000 steps. The trace files of 10 longer runs were combined using Tracer v1.6 (Rambaut et al., 2018) to calculate mean migration with a burn‐in of 50,000. We used the migration rates of the two weevils as measures of gene flow.

### 2.5 Analysis of the influence of geographic and climatic factors on genetic variation

Geographic and climatic effects on population genetic variation were examined with two methods. First, the Mantel test was used to test the presence of isolation by distance (IBD) and isolation by environment (IBE) by correlating pairwise genetic differentiation (estimated as *F*_ST_/(1−*F*_ST_)) with geographic distance (km) and climate data using the R package *ade4* v 1.7‐15, with 10,000 permutations (Figure S1). Nineteen climate variables for each collection site of the two species were obtained from the WorldClim database (https://worldclim.org/, last accessed on 28, December 2020). The standard (19) WorldClim Bioclimatic variables for WorldClim v2 at a 10‐min spatial resolution were used.

Second, multivariate redundancy analysis (RDA) was used to examine the variance of microsatellite genotypes which can be explained by climate and geography and their collinear part (spatial autocorrelation climate change). RDA is the PCA analysis of the fitted value matrix of the multiple linear regression between the variable matrix and the explanatory variable matrix (Forester et al., 2018). Nineteen climate variables were downloaded as described above. We converted the matrices of pairwise geographic distances into principal components of neighborhood matrices (PCNM) using the R package function *pcnm* in *vegan* v2.5‐6 (https://github.com/vegandevs/vegan, last accessed on 28, December 2020). The first half of the positive feature vector as the explanatory variable of the population structure was kept. To avoid high linearity among the PCNM and climate variables, we excluded variables with a variance inflation factor (VIF) exceeding 15 calculated in *vegan* v2.5‐6. We used the R package *vegan* v2.5‐6 to analyze the full model (environment and geography) and partial model (environment or geography) of RDA. The independent environmental effect was calculated by constraining the effect of geography, while the independent geographic effect was calculated by constraining the effect of the environment in a partial RDA analysis. The collinearity effect was calculated by subtracting the independent effects of environment and geography from the total variance explained in the full RDA analysis.

## 3 RESULTS

### 3.1 Population genetic diversity

The null allele frequency and Hardy–Weinberg equilibrium (HWE) of loci scored for TTW and TRW are shown in Tables S2 and S3. For TTW, the observed heterozygosity (*H*_O_) for each population ranged from 0.15 to 0.36. The inbreeding coefficient (*F*_IS_) ranged from −0.24 to 0.31, with an average value of 0.06; the polymorphism information content of each population (*PIC*) ranged from 0.15 to 0.30; the allelic richnesses (*A*_R_) of six populations were not quite significantly different, varying from 1.50 in LNDL to 2.47 in SDRZ. The private allelic richness was also low for the 13 populations. The standardized total number of alleles (*A*_S_) ranged from 20.70 in NXLW to 42.03 in SDRZ. The standardized expected heterozygosity (*H*_ES_) varied from 0.28 in BJSY to 0.69 in TJTJ (Table 2). Population differentiation (as measured by *F*_ST_) ranged from 0.0197 to 0.6650 (Table 3).

**TABLE 2:** Parameters of genetic diversity in populations of *Eucryptorrhynchus brandti* (TTW)

Population	*N*	*A*_T_	*A*_S_	*A*_R_	*P*_AR_	*H*_O_	*H*_ET_	*H*_ES_	*F*_IS_
BJHD	13	45	28.46	1.57	0.07	0.15	0.43	0.42	0.18
BJHR	12	42	32.15	1.74	0.14	0.22	0.57	0.57	0.13
BJSY	20	35	21.87	1.57	0.00	0.16	0.29	0.28	0.08
BJYQ	20	54	29.36	1.75	0.10	0.23	0.47	0.46	0.01
HBJZ	5	35	35.00	1.97	0.27	0.24	0.59	0.59	0.20
HNZZ	20	39	26.19	1.72	0.03	0.30	0.40	0.40	−0.24
LNDL	10	39	32.03	1.50	0.02	0.18	0.56	0.56	0.01
NXLW	12	24	20.70	1.97	0.24	0.35	0.31	0.31	−0.13
NXPL	13	27	21.08	1.80	0.15	0.22	0.32	0.32	0.14
NXZW	13	45	33.15	2.02	0.19	0.22	0.58	0.57	0.31
SDRZ	16	68	42.03	2.47	0.36	0.29	0.71	0.70	0.25
SDTA	12	39	25.63	1.72	0.02	0.26	0.36	0.35	−0.18
TJTJ	14	63	40.88	2.28	0.45	0.36	0.69	0.69	−0.06

Abbreviations: *A*_R_, average allelic richness (for 5 specimens); *A*_S_, standardized total number of alleles for 5 specimens per sample. *H*_ET_, expected heterozygosity; *A*_T_, total number of alleles; *F*_IS_, inbreeding coefficient; *H*_ES_, standardized expected heterozygosity (for 5 specimens); N, Sample size; *P*_AR_, private allelic richness (for 5 specimens).

**TABLE 3:** Pairwise *F*_ST_ among 13 populations of *Eucryptorrhynchus brandti* (TTW)

Populations	BJHD	BJHR	BJSY	BJYQ	HBJZ	HNZZ	LNDL	NXLW	NXPL	NXZW	SDRZ	SDTA
BJHR	0.3445											
BJSY	0.6170	0.6034										
BJYQ	0.5628	0.5456	0.5054									
HBJZ	0.4981	0.4551	0.4013	0.2560								
HNZZ	0.5333	0.5186	0.3740	0.2679	0.1276							
LNDL	0.5972	0.5586	0.0417	0.4989	0.3958	0.3747						
NXLW	0.3886	0.1114	0.6256	0.5629	0.4671	0.5417	0.5723					
NXPL	0.0233	0.3132	0.5463	0.5104	0.4069	0.4483	0.5041	0.3614				
NXZW	0.2859	0.1357	0.5563	0.5066	0.3996	0.4669	0.5105	0.1431	0.2666			
SDRZ	0.3997	0.3745	0.3216	0.1234	0.0197	0.1184	0.2876	0.3918	0.3274	0.3496		
SDTA	0.4532	0.0499	0.6650	0.5969	0.5271	0.5791	0.6173	0.0783	0.4148	0.2052	0.4212	
TJTJ	0.3476	0.3616	0.2645	0.2243	0.0820	0.1008	0.2587	0.3936	0.2849	0.3123	0.0658	0.4356

Note

All values are different significantly.

For TRW, the observed heterozygosity (*H*_O_) for each population ranged from 0 to 0.17. The inbreeding coefficient (*F*_IS_) ranged from −0.14 to 0.78, with an average value of 0.43; the polymorphism information content of each population (*PIC*) ranged from 0.10 to 0.23; the allelic richness (*A*_R_) of 11 populations was not significantly different, varying from 1.30 in BJCY to 1.84 in SDRZ. Private allelic richness was also low for the 11 populations. The standardized total number of alleles (*A*_S_) ranged from 14.00 in SXYC to 35.51 in BJSY. The standardized expected heterozygosity (*H*_ES_) varied from 0.13 in SXYC to 0.67 in BJSY (Table 4). The *F*_ST_ values between populations ranged from −0.0724 to 0.6845 (Table 5).

**TABLE 4:** Parameters of genetic diversity in populations of *Eucryptorrhynchus scrobiculatus* (TRW)

Population	*N*	*A*_T_	*A*_S_	*A*_R_	*P*_AR_	*H*_O_	*H*_ET_	*H*_ES_	*F*_IS_
BJCY	5	24	24.00	1.30	0.03	0.04	0.35	0.35	0.77
BJHD	13	21	17.21	1.75	0.41	0.17	0.28	0.27	0.40
BJHR	13	25	19.53	1.43	0.09	0.16	0.30	0.29	0.02
BJSY	10	44	35.51	1.82	0.25	0.06	0.68	0.67	0.78
LNDL	7	37	32.76	1.65	0.19	0.07	0.64	0.63	0.69
NXLW	12	23	16.86	1.35	0.04	0.16	0.20	0.20	−0.11
NXPL	13	45	30.89	1.49	0.10	0.12	0.54	0.52	0.22
NXZW	13	20	14.46	1.39	0.08	0.00	0.15	0.14	1.00
SDRZ	15	46	31.98	1.84	0.33	0.10	0.63	0.60	0.63
SXYC	5	14	14.00	1.36	0.03	0.08	0.13	0.13	0.41
SXYL	12	20	16.56	1.36	0.03	0.17	0.24	0.23	−0.14

Abbreviations: *A*_R_, average allelic richness (for 5 specimens); *A*_T_, total number of alleles; *A*_S_, standardized total number of alleles for 5 specimens per sample; *F*_IS_, inbreeding coefficient; *H*_ET_, expected heterozygosity; *H*_ES_, standardized expected heterozygosity (for 5 specimens); N, Sample size; *P*_AR_, private allelic richness (for 5 specimens).

**TABLE 5:** Pairwise *F*_ST_ among 11 populations of *Eucryptorrhynchus scrobiculatus* (TRW)

Population	BJCY	BJHD	BJHR	BJSY	LNDL	NXLW	NXPL	NXZW	SDRZ	SXYC
BJHD	0.5886									
BJHR	0.6359	0.5803								
BJSY	0.2108	0.4959	0.5167							
LNDL	0.1069	0.5074	0.5424	−0.0724						
NXLW	0.6462	0.6240	0.6068	0.5121	0.5613					
NXPL	0.5125	0.5406	0.4079	0.3910	0.3963	0.4482				
NXZW	0.7476	0.6034	0.5333	0.6629	0.7035	0.6551	0.6732			
SDRZ	0.1713	0.5189	0.5020	0.0262	−0.0442	0.5082	0.3697	0.6723		
SXYC	0.1927	0.5864	0.6789	0.2406	0.2261	0.6783	0.5620	0.7548	0.3196	
SXYL	0.6681	0.6234	0.6176	0.5073	0.5677	−0.0127	0.4637	0.6639	0.5179	0.6845

Note

All values are different significantly.

### 3.2 Population genetic structure

**FIGURE 2:**
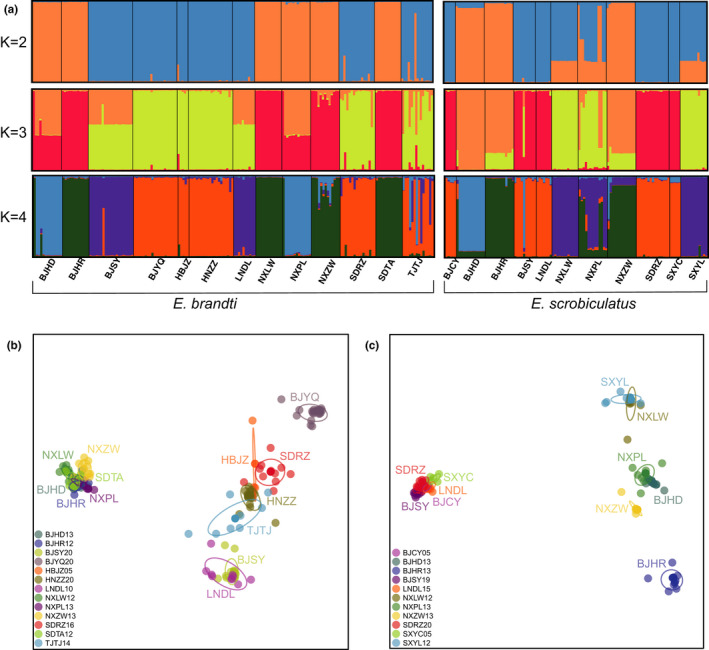
Genetic structure of *Eucryptorrhynchus brandti* (TTW) and *E*. *scrobiculatus* (TRW) populations. (a) Genetic structure inferred from STRUCTURE with *k* = 2, 3, 4. Genetic structure of TTW (b) and TRW (c) populations based on 10 markers using DAPC. Codes for the populations are shown in Figure 1

Structure analysis showed that the optimal K was two for TTW and three for TRW. For TTW, one cluster is composed of individuals from three geographically distant provinces and include BJHD, BJHR, NXLW, NXPL, NXZW, and SDTA (Figure 2a). Individuals from four geographically distant provinces and including BJSY, BJYQ, HBJZ, HNZZ, and LNDL were all assigned to the second cluster. Individuals of SDRZ and TJTJ showed an admixture of the two clusters, with the second cluster as the dominant one. When the number of clusters increased to 4, the level of admixture increased mainly in TJTJ, SDRZ, NXZW, and genetic differentiation of the overall population remained consistent (Figure 2a).

For TRW, genetic mixing in populations was detected; this was the case for individuals from BJHR, NXPL, and NXZW (Figure 2a). In addition, the other TRW populations were divided into three clusters. One cluster was composed of individuals from BJHD. The second cluster was composed of individuals from two provinces with a smaller distance between SXYL and NXLW. The last cluster was found in populations of BJSY, BJCY, LNDL, SDRZ, and SXYC from four provinces. One individual from BJSY showed an obvious effect of gene flow. When the number of clusters increased to 4, a higher level of mixing was evident among the geographic populations (Figure 2a).

Discriminant analysis of principal component showed similar patterns of population differentiation, with two geographically distant population clusters for both TTW and TRW although the second cluster was more diffused (Figure 2b and c). Individuals from different geographic regions were genetically divided into clusters similar to those identified from the STRUCTURE analysis.

Overall, population genetic structure analysis showed that some populations from different geographic locations could be assigned to the same genetic cluster (e.g., BJHD, BJHR, NXLW, NXPL, NXZW, and SDTA in TTW, see Figure 1), while some populations collected from the same area could be assigned to different groups (e.g., BJHR and BJHD in TRW, see Figure 1). Additionally, individuals from the same population could be assigned to different clusters (e.g., TJTJ in TTW and NXZW in TRW).

### 3.3 Gene flow and demographic history

**FIGURE 3:**
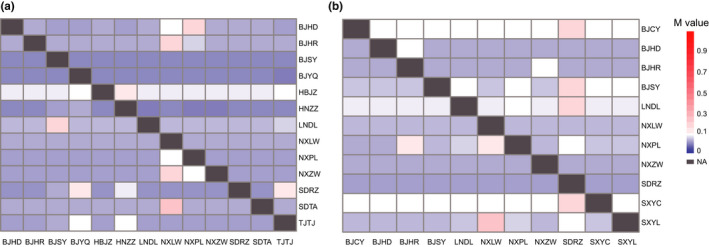
Recent gene flow among populations of *Eucryptorrhynchus brandti* (TTW) (a) and *E*. *scrobiculatus* (TRW) (b) inferred from BAYESASS. Migration rate (*M* value) of TTW ranges from 0.0100–0.8782; migration rate of TRW ranges from 0.0128–0.8710

Gene flow estimates in the two weevils ranged from 0.0097 to 0.0170 (95% confidence intervals) in TTW (Figure 3a, Table S4) and from 0.0123 to 0.0389 (95% C.I.s) in TRW (Figure 3b, Table S5). For TTW, low gene flow was detected between HBJZ and other populations. High levels of gene flow were found between BJHD and BJPL, BJHR and NXLW, LNDL and BJSY, NXZW and NXLW, and SDTA and NXLW. For TRW, BJCY, SXYC, and LNDL had low gene flow with other populations. High levels of gene flow were found between BJCY, BJSY, LNDL and SDRZ, BJHR, NXLW, and NXPL, and between NXLW and SXYL.

### 3.4 Geographic and climate impacts on genetic variation

Mantel tests showed that there was no significant correlation between genetic distance and geographic distance (TTW, *p* = .47, *r* = −0.01; TRW, *p* = .15, *r* = 0.14), or environmental conditions (TTW, *p* = .67, *r* = −0.05; TRW, *p* = .27, *r* = 0.09; Figure S1).

**FIGURE 4:**
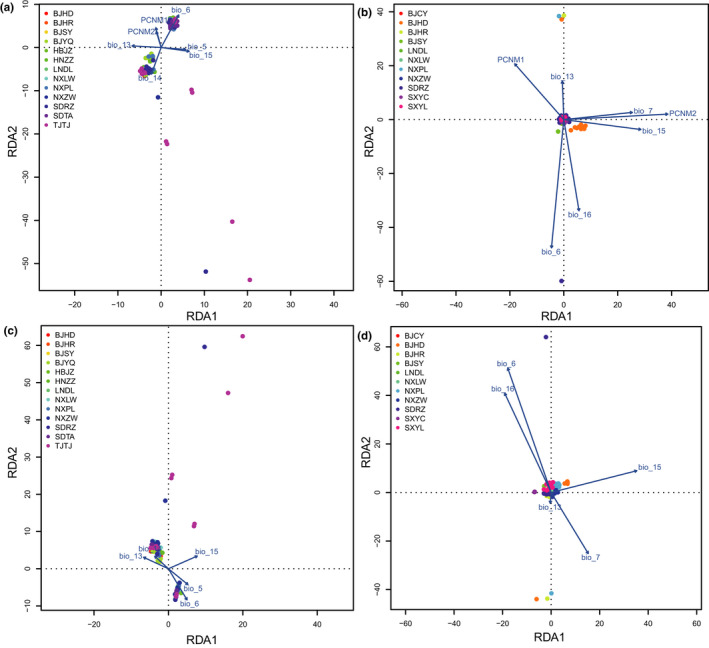
RDA analysis on genetic variance of *Eucryptorrhynchus brandti* (TTW) and *E*. *scrobiculatus* (TRW) explained by the environmental effects of climate and geography. A full RDA model was run by considering environmental and geographic effects simultaneously (a, TTW; c, TRW), and a partial RDA model was run by constraining geographic effects to analyze the correlation of environmental variables (b, TTW; d, TRW). Individuals from the same population are indicated by circles with the same color. PCNM1‐2, geographic variables; the highest temperature of the warmest month (bio5), Min temperature of coldest month (bio6), temperature annual range (bio7), precipitation of wettest month (bio13), precipitation of driest month (bio14), precipitation seasonality (coefficient of variation) (bio15) and precipitation of wettest quarter (bio16), climate variables. Correlations of each variable are indicated by an arrow. Long arrows indicate a high correlation between the variable and genetic distance

For TTW, effects of environmental conditions and geography accounted for 93.94% and 29.70% of the explained genetic variance, respectively, while however, there is no collinear component between them (Table S6). When considering both environmental and geographic effects in the RDA analysis, five climatic variables (the highest temperature of the warmest month [bio5], Min Temperature of Coldest Month [bio6], Precipitation of Wettest Month [bio13], Precipitation of Driest Month [bio14] and Precipitation Seasonality (Coefficient of Variation) [bio15]) and two geographic variables (PCNM1 and PCNM2) were related with genetic distance (Figure 4a). When geographic variables were controlled in the RDA analysis, bio5, bio6, bio13, bio14, and bio15 were correlated with genetic distance (Figure 4c).

For TRW, effects of environmental conditions and geography accounted for 41.90% and 55.73% of the explained genetic variance, respectively, while their collinear component accounted for 2.38% (Table S6). When considering both environmental and geographic impacts in the RDA analysis, five climatic variables (Min Temperature of Coldest Month [bio6], Temperature Annual Range [bio7], Precipitation of Wettest Month [bio13], Precipitation Seasonality (Coefficient of Variation) [bio15] and Precipitation of Wettest Quarter[bio16]) and two geographic variables (PCNM1 and PCNM2) were related to genetic distance (Figure 4b). When geographic variables were restricted in the RDA analysis, bio6, bio7, bio13, bio15, and bio16 were correlated with genetic distance (Figure 4d).

## 4 DISCUSSION

### 4.1 Dispersal of TTW and TRW in China

The distribution range of *A*. *altissima* matches the current distribution range of the two weevils, with all three taxa mainly distributed in Northeast, North, Northwest, and Central South China. However, in some southern provinces, such as Hubei and Sichuan, *A*. *altissima* trees grow sporadically in rural areas, and their density is extremely low. For regions in Northeast, North China, and Northwest China, such as Shandong, Ningxia, and Henan, *A*. *altissima* is typically planted as a street tree, with reductions in vigor due to the transplanting process contributing to particularly high levels of damage from the two weevils.

*Ailanthus altissima* may have originated from the Indian subcontinent, first spreading to Tibet with the collision of plates, and then spreading to other regions such as East Asia, Europe, and even North America via Tibet (Su et al., 2021). In addition, the humid and sub‐humid areas of the Qinghai‐Tibet Plateau provide a suitable climate for *A*. *altissima* (Yan, 2019). Perhaps this climate region provides a source location for the two weevils, although samples were unavailable from this region.

Population genetics analysis can provide new ideas for routes of spread of species (Boissin et al., 2012a; Estoup & Guillemaud, 2010). Based on the STRUCTURE and DAPC analyses, the two weevils show a clear population genetic structure across China. The populations of TTW and TRW can be divided into two and three clusters, respectively, while there is evidence of admixture in both weevils (Figures 2 and S2). Some geographically distant populations were grouped and there was no IBD in either TTW or TRW, with results also indicating that high levels of genetic diversity and gene flow were evident in well‐separated populations. According to information from the China Forestry and Grassland Administration (http://www.forestry.gov.cn/), TTW and TRW can be spread through seedling transfer. This suggests migration of TTW and TRW mainly passively through human activities due to seedling transfer. Similar passive and human‐assisted ways of spread occur in other pests, such as the *Frankliniella occidentalis* (Cao et al., 2017) and *Aedes aegypti* (Eugenio et al., 2015).

In our study, TTW and TRW provide a pattern of cross‐diffusion between the northeast and southwest regions. According to the 2014–2017 National Forestry Pest Survey Results (Song, 2020), TTW and TRW are distributed in northeast, northwest, and southeast China. However, we found that population diversity in some southeast distribution areas is lower than that of northwest and north China. This might be caused by a lower population density in some southern populations which could account for sampling difficulties at some of our locations (e.g., we only collected five TTW individuals in HBJZ).

### 4.2 The influence of climate and geographic factors on population genetic variation

The results of IBD and IBE suggest that the genetic distance of two species has no strong association with geographic distance or climate factors. Since the IBE analysis considered the correlation between two matrices using a Mantel test and reduced the dimensions of the original data, we conducted a multivariate RDA analysis. The results showed that the variation in genetic distance between samples may have some association to climate factors in both the TTW and TRW populations. Among these, temperature and precipitation may affect patterns of genetic variation in the two weevils. The importance of these variables has previously been noted for another pest, the melon thrips *Thrips palmi* (Cao et al., 2019).

### 4.3 Implications for pest management and usage in biological control of invasive plant

The integration of DNA data and pest risk assessment offers important monitoring tools for evaluating the relative success of pest invasions (Stepien et al., 2005). Northwest China (including the Ningxia Hui Autonomous Region, Qinghai, Shanxi Province, etc.) has classified TTW and TRW as key prevention and quarantine targets in recent years. The host plant of TTW and TRW, the tree‐of‐heaven, is a plant that tolerates a wide range of climatic conditions and can be used when planted as seedlings to construct shelterbelts in northern and northwestern China (Hu, 1979). Our results point to high levels of differentiation among some populations but low genetic diversity, consistent with spread through human activities and a buildup of the pest from low initial numbers. Due to the weak dispersal capacity of TTW and TRW, quarantine measures and disinfestation should be effective and used to limit transmission during seedling transfer between Chinese provinces and cities. Such measures should prevent the two species of weevil from spreading further.

In the United States, *A*. *altissima* has been listed as an invasive weed because it has destroyed the landscape of urban areas in recent years. The two weevils studied here are monophagous wood‐boring pests which only feed on *A*. *altissima*. The latitude and climatic conditions of the United States and China are similar, and there is a substantial area suitable for these two weevils in the United States (Ji et al., 2017). We propose that these two weevils could be spread through humans to act as biological control agents in controlling this tree.

## 5 CONCLUSION

In our study, we used microsatellite markers to explore the population genetic diversity and structure of two *Eucryptorrhynchus* weevils. We found that populations of both TTW and TRW have high levels of differentiation but low genetic diversity. The patterns of genetic structure and intraspecific admixture point to the human‐mediated dispersal in these two weevils. We found that temperature and precipitation may indirectly associate with genetic differentiation in the two weevils. The results provide information for integrated pest management of these two pest weevils in China and their use as biological control agents in other continents.

## CONFLICT OF INTEREST

There are no conflicts of interest.

## AUTHOR CONTRIBUTION

**Yu‐Jie Zhang:** Data curation (equal); Formal analysis (equal); Resources (lead); Writing – original draft (equal). **Wei Song:** Formal analysis (equal); Writing – original draft (equal). **Li‐Jun Cao:** Data curation (equal); Formal analysis (equal). **Jin‐Cui Chen:** Resources (equal). **Ary A. Hoffmann:** Writing – review & editing (equal). **Junbao Wen:** Funding acquisition (equal); Project administration (equal); Writing – review & editing (equal). **Shu‐Jun Wei:** Conceptualization (equal); Writing – original draft (equal); Writing – review & editing (equal).

## Supporting information

Supplementary Material

## Data Availability

Microsatellite data generated in this study were deposited to the Dryad data repository (https://doi.org/10.5061/dryad.vx0k6djtr).
