# Supplementary material for: Population differentiation and intraspecific genetic admixture in two Eucryptorrhynchus weevils (Coleoptera: Curculionidae) across northern China
Source: Ecol Evol. 2022 Apr 6;12(4):e8806. doi: 10.1002/ece3.8806 (PMC8986550; doi:10.1002/ece3.8806)
Supplement: Supplementary file 1 — Supplementary Material [file ECE3-12-e8806-s001.docx]

**Table S1** Information on the microsatellite loci used in this study.

| Locus | Dye | Primer sequences（5’– 3’） | Primer sequences（5’– 3’） | Motifs | size (bp) | Tm (°C) |
| --- | --- | --- | --- | --- | --- | --- |
| S01 | HEX | GGTGGAACATCACGCAAACA | TGCGCGTTGATTATCTTTCAGA | (AT)6 | 95 | 56 |
| S07 | HEX | AGGCGTTATTAGTGGGTGGA | CATCCCTGTGTTGTGCTTCC | (AG)7 | 140 | 56 |
| S09 | HEX | GTATTAGTGGCAACGTCGGATT | CTTCGCGGCGAGCCAAAG | (AT)5 | 140 | 56 |
| S12 | HEX | CTCCAAGCGCTCGATGTCTT | CGACAGCTCGGAAATGATCG | (AAG)7 | 142 | 56 |
| S18 | HEX | CAAGACACGCCTTGGAAACA | ATCCGAAGTCAGAGTGGTCG | (AT)5 | 173 | 56 |
| S19 | HEX | CCGAGGCTTCTGTTAACGTC | TCCTGTACCTCGTTACGTCA | (AG)5 | 179 | 56 |
| S27 | FAM | GGATACACGATTCCTATCCAGT | CGATAGGTTCCAGTTGCAGG | (AAAT)5 | 191 | 56 |
| S38 | FAM | TGGACTACAGTGTCAAGGCG | AGGACTGGTTGATGGGAGTC | (AC)11 | 243 | 56 |
| S40 | FAM | CGGAGCTCAAGGTAATCGAC | GAGACGGATTTCTCAAGGGC | (AT)5 | 251 | 56 |
| S60 | ROX | AAACCTGACACAGAGGAGGC | GGAGGATCGACGCCTTTAGA | (AT)5 | 349 | 56 |

**Table S2 Null allele frequency and Hardy-Weinberg equilibrium (HWE) of *Eucryptorrhynchus brandti* (TTW)**

| Sites | HWE | | | | | | | | | | Null Allele Frequencies | | | | | | | | | |
| --- | --- | --- | --- | --- | --- | --- | --- | --- | --- | --- | --- | --- | --- | --- | --- | --- | --- | --- | --- | --- |
|  | S01 | S07 | S09 | S12 | S18 | S19 | S27 | S38 | S40 | S60 | S01 | S07 | S09 | S12 | S18 | S19 | S27 | S38 | S40 | S60 |
| BJHD | NA | NA | 0.54 | NA | 0.05 | 0.04 | 0.23 | 0.04 | NA | NA | 0.00 | NA | 0.07 | NA | 0.00 | 0.18 | 0.00 | 0.18 | NA | NA |
| BJHR | NA | NA | 1.00 | NA | 1.00 | 0.31 | 0.92 | 0.01 | 1.00 | NA | NA | NA | 0.00 | NA | 0.00 | 0.11 | 0.00 | 0.23 | 0.00 | NA |
| BJSY | NA | NA | 0.00 | NA | NA | 1.00 | 0.98 | 0.01 | 0.03 | NA | NA | NA | 0.02 | NA | NA | 0.92 | 0.00 | 0.18 | 0.15 | NA |
| BJYQ | NA | NA | 0.28 | NA | 0.00 | NA | 0.39 | 0.00 | 0.14 | NA | 0.00 | NA | 0.09 | NA | 0.00 | NA | 0.00 | 0.20 | 0.00 | NA |
| HBJZ | NA | 0.11 | 0.24 | NA | 0.02 | NA | 1.00 | NA | NA | NA | NA | 0.07 | 0.00 | NA | 0.29 | NA | 0.00 | NA | NA | NA |
| HNZZ | NA | 0.00 | 0.65 | NA | 1.00 | NA | 0.86 | 0.08 | NA | NA | 0.00 | 0.00 | 0.00 | NA | 0.00 | NA | 0.00 | 0.13 | NA | NA |
| LNDL | NA | NA | 0.52 | NA | NA | 0.11 | 0.08 | 0.03 | NA | NA | NA | NA | 0.00 | NA | NA | 0.89 | 0.00 | 0.20 | NA | NA |
| NXLW | NA | NA | 0.30 | NA | 0.32 | NA | 1.00 | 0.00 | NA | 1.00 | NA | NA | 0.19 | NA | 0.00 | NA | 0.00 | 0.00 | NA | 0.68 |
| NXPL | 0.04 | NA | 1.00 | NA | 0.37 | 0.04 | 1.00 | 0.12 | 0.39 | NA | 0.28 | NA | 0.00 | NA | 0.00 | 0.18 | 0.00 | 0.15 | 0.10 | 0.96 |
| NXZW | NA | NA | 1.00 | 0.00 | 0.06 | NA | 0.88 | 1.00 | 0.00 | 0.17 | NA | NA | 0.00 | 0.76 | 0.01 | NA | 0.00 | 0.00 | 0.26 | 0.84 |
| SDRZ | NA | 0.00 | 0.01 | NA | 0.25 | NA | 0.01 | 0.00 | 0.04 | NA | NA | 0.06 | 0.14 | NA | 0.07 | 0.94 | 0.13 | 0.21 | 0.18 | NA |
| SDTA | NA | NA | 0.54 | NA | 1.00 | NA | 0.77 | 0.00 | NA | NA | NA | NA | 0.01 | NA | 0.00 | NA | 0.00 | 0.00 | NA | NA |
| TJTJ | NA | 0.05 | 0.00 | NA | 0.50 | NA | 0.18 | 0.35 | 0.20 | NA | NA | 0.13 | 0.00 | NA | 0.00 | NA | 0.00 | 0.11 | 0.13 | NA |

**Table S3 Null allele frequency and Hardy-Weinberg equilibrium (HWE) of *Eucryptorrhynchus scrobiculatus* (TRW)**

| Sites | HWE | | | | | | | | | | Null Allele Frequencies | | | | | | | | | |
| --- | --- | --- | --- | --- | --- | --- | --- | --- | --- | --- | --- | --- | --- | --- | --- | --- | --- | --- | --- | --- |
|  | S01 | S07 | S09 | S12 | S18 | S19 | S27 | S38 | S40 | S60 | S01 | S07 | S09 | S12 | S18 | S19 | S27 | S38 | S40 | S60 |
| BJCY | NA | 1 | 0.05 | NA | NA | 0.05 | NA | NA | NA | NA | NA | 0.45 | 0.33 | NA | NA | 0.77 | NA | NA | NA | NA |
| BJHD | 1.00 | 0.26 | 0.04 | 1.00 | 0.60 | 0.04 | 0.04 | 0.04 | 0.00 | NA | 0.00 | 0.48 | 0.18 | 0.00 | 0.00 | 0.18 | 0.17 | 0.18 | 0.25 | NA |
| BJHR | 1.00 | 0.51 | NA | 1.00 | 1.00 | NA | NA | NA | 1 | NA | 0.00 | 0.39 | NA | 0.00 | 0.00 | 0.00 | NA | NA | 0.00 | NA |
| BJSY | NA | 0.01 | NA | 0.05 | 0.09 | 0.01 | 0.07 | 0.00 | 0.05 | NA | NA | 0.71 | NA | 0.28 | 0.24 | 0.47 | 0.18 | 0.37 | 0.20 | NA |
| LNDL | NA | 0.02 | NA | NA | 1.00 | 0.11 | 0.08 | 0.05 | NA | NA | NA | 0.71 | NA | NA | 0.05 | 0.45 | 0.19 | 0.33 | 0.00 | NA |
| NXLW | NA | 0.01 | NA | NA | 1.00 | NA | NA | 0.01 | NA | NA | 0.00 | 0.41 | 0.00 | NA | 0.00 | NA | NA | 0.00 | NA | NA |
| NXPL | 1.00 | NA | NA | NA | 0.01 | NA | NA | 0.01 | 1.00 | NA | 0.00 | NA | NA | NA | 0.01 | NA | NA | 0.25 | 0.00 | NA |
| NXZW | 0.04 | 0.00 | NA | 0.04 | NA | 0.01 | NA | 0.04 | 0.04 | NA | 0.18 | 0.48 | NA | 0.18 | NA | 0.24 | NA | 0.19 | 0.18 | NA |
| SDRZ | NA | 0.00 | 0.00 | NA | 1.00 | NA | 0.00 | 0.00 | 0.07 | NA | 0.00 | 0.76 | 0.23 | NA | 0.00 | NA | 0.31 | 0.37 | 0.13 | NA |
| SXYC | NA | NA | NA | NA | 1.00 | NA | NA | 0.11 | NA | NA | NA | 0.00 | NA | 0.00 | 0.06 | NA | NA | 0.27 | NA | NA |
| SXYL | NA | 1.00 | NA | NA | 0.59 | NA | NA | 0.05 | NA | NA | 0.00 | 0.00 | NA | 0.00 | 0.05 | NA | NA | 0.00 | NA | NA |

**Table S4** Estimates of recent gene flow (M value from BAYESASS analysis) in 13 populations of *Eucryptorrhynchus brandti* (TTW)

M values of TTW:

| TTW | BJHD | BJHR | BJSY | BJYQ | HBJZ | HNZZ | LNDL | NXLW | NXPL | NXZW | SDRZ | SDTA | TJTJ |
| --- | --- | --- | --- | --- | --- | --- | --- | --- | --- | --- | --- | --- | --- |
| BJHD | 0.6802 | 0.0132 | 0.0129 | 0.0128 | 0.0132 | 0.0128 | 0.0132 | 0.0256 | 0.1639 | 0.0131 | 0.0132 | 0.0132 | 0.0128 |
| BJHR | 0.0134 | 0.6801 | 0.0133 | 0.0133 | 0.0133 | 0.0134 | 0.0133 | 0.1697 | 0.0167 | 0.0133 | 0.0134 | 0.0134 | 0.0134 |
| BJSY | 0.0101 | 0.0101 | 0.8782 | 0.0102 | 0.0101 | 0.0105 | 0.0102 | 0.0101 | 0.0101 | 0.0101 | 0.0101 | 0.0101 | 0.0101 |
| BJYQ | 0.0102 | 0.0101 | 0.0102 | 0.8782 | 0.0101 | 0.0106 | 0.0101 | 0.0101 | 0.0101 | 0.0101 | 0.0101 | 0.0101 | 0.01 |
| HBJZ | 0.0186 | 0.0186 | 0.0185 | 0.021 | 0.6851 | 0.0895 | 0.0187 | 0.0185 | 0.0186 | 0.0187 | 0.0185 | 0.0186 | 0.0371 |
| HNZZ | 0.0101 | 0.0101 | 0.0121 | 0.0137 | 0.0102 | 0.8733 | 0.01 | 0.0101 | 0.01 | 0.01 | 0.0101 | 0.0101 | 0.0101 |
| LNDL | 0.0145 | 0.0146 | 0.1569 | 0.0145 | 0.0145 | 0.0146 | 0.6813 | 0.0145 | 0.0146 | 0.0145 | 0.0145 | 0.0145 | 0.0166 |
| NXLW | 0.0133 | 0.0134 | 0.0134 | 0.0134 | 0.0133 | 0.0134 | 0.0132 | 0.84 | 0.0133 | 0.0133 | 0.0133 | 0.0133 | 0.0133 |
| NXPL | 0.0128 | 0.0128 | 0.0128 | 0.0129 | 0.0128 | 0.0127 | 0.0128 | 0.0257 | 0.8334 | 0.0127 | 0.0128 | 0.0129 | 0.0128 |
| NXZW | 0.0127 | 0.0127 | 0.0129 | 0.0128 | 0.0128 | 0.0128 | 0.0128 | 0.1672 | 0.0255 | 0.6795 | 0.0127 | 0.0129 | 0.0129 |
| SDRZ | 0.0116 | 0.0117 | 0.0133 | 0.1177 | 0.0115 | 0.0183 | 0.0116 | 0.0115 | 0.0115 | 0.0116 | 0.6784 | 0.0115 | 0.0798 |
| SDTA | 0.0132 | 0.0133 | 0.0134 | 0.0135 | 0.0134 | 0.0133 | 0.0134 | 0.1727 | 0.0135 | 0.0134 | 0.0134 | 0.68 | 0.0135 |
| TJTJ | 0.0125 | 0.0124 | 0.0133 | 0.0493 | 0.0124 | 0.062 | 0.0123 | 0.0124 | 0.0123 | 0.0123 | 0.0124 | 0.0124 | 0.764 |

95% confidence intervals of M value of TTW:

| TTW | BJHD | BJHR | BJSY | BJYQ | HBJZ | HNZZ | LNDL | NXLW | NXPL | NXZW | SDRZ | SDTA | TJTJ |
| --- | --- | --- | --- | --- | --- | --- | --- | --- | --- | --- | --- | --- | --- |
| BJHD | 0.0130 | 0.0127 | 0.0124 | 0.0123 | 0.0126 | 0.0122 | 0.0127 | 0.0172 | 0.0325 | 0.0126 | 0.0127 | 0.0128 | 0.0122 |
| BJHR | 0.0128 | 0.0129 | 0.0128 | 0.0128 | 0.0129 | 0.0128 | 0.0128 | 0.0335 | 0.0163 | 0.0128 | 0.0129 | 0.0128 | 0.0129 |
| BJSY | 0.0098 | 0.0099 | 0.0276 | 0.0099 | 0.0099 | 0.0102 | 0.0098 | 0.0098 | 0.0098 | 0.0099 | 0.0098 | 0.0098 | 0.0099 |
| BJYQ | 0.0099 | 0.0098 | 0.0099 | 0.0278 | 0.0098 | 0.0103 | 0.0098 | 0.0098 | 0.0098 | 0.0098 | 0.0098 | 0.0098 | 0.0098 |
| HBJZ | 0.0177 | 0.0175 | 0.0175 | 0.0198 | 0.0175 | 0.0346 | 0.0176 | 0.0174 | 0.0176 | 0.0177 | 0.0174 | 0.0177 | 0.0240 |
| HNZZ | 0.0097 | 0.0098 | 0.0115 | 0.0132 | 0.0099 | 0.0288 | 0.0097 | 0.0098 | 0.0097 | 0.0098 | 0.0098 | 0.0098 | 0.0098 |
| LNDL | 0.0140 | 0.0139 | 0.0342 | 0.0139 | 0.0139 | 0.0139 | 0.0140 | 0.0139 | 0.0139 | 0.0138 | 0.0138 | 0.0139 | 0.0159 |
| NXLW | 0.0127 | 0.0128 | 0.0128 | 0.0130 | 0.0128 | 0.0129 | 0.0127 | 0.0324 | 0.0128 | 0.0128 | 0.0129 | 0.0126 | 0.0128 |
| NXPL | 0.0122 | 0.0123 | 0.0124 | 0.0124 | 0.0123 | 0.0122 | 0.0124 | 0.0172 | 0.0320 | 0.0122 | 0.0124 | 0.0123 | 0.0123 |
| NXZW | 0.0121 | 0.0123 | 0.0125 | 0.0123 | 0.0123 | 0.0123 | 0.0122 | 0.0403 | 0.0298 | 0.0124 | 0.0123 | 0.0124 | 0.0124 |
| SDRZ | 0.0113 | 0.0113 | 0.0127 | 0.0360 | 0.0112 | 0.0245 | 0.0113 | 0.0110 | 0.0112 | 0.0112 | 0.0115 | 0.0111 | 0.0263 |
| SDTA | 0.0128 | 0.0128 | 0.0129 | 0.0128 | 0.0129 | 0.0129 | 0.0128 | 0.0326 | 0.0129 | 0.0129 | 0.0129 | 0.0128 | 0.0128 |
| TJTJ | 0.0120 | 0.0120 | 0.0128 | 0.0261 | 0.0119 | 0.0284 | 0.0119 | 0.0120 | 0.0118 | 0.0118 | 0.0118 | 0.0119 | 0.0291 |

**Table S5** Estimates of recent gene flow (M value from BAYESASS analysis) in 11 populations of *Eucryptorrhynchus scrobiculatus* (TRW)

M values of TRW:

| TRW | BJCY | BJHD | BJHR | BJSY | LNDL | NXLW | NXPL | NXZW | SDRZ | SXYC | SXYL |
| --- | --- | --- | --- | --- | --- | --- | --- | --- | --- | --- | --- |
| BJCY | 0.6876 | 0.021 | 0.0209 | 0.0209 | 0.0208 | 0.0209 | 0.0208 | 0.0209 | 0.1245 | 0.0208 | 0.0208 |
| BJHD | 0.014 | 0.8474 | 0.0275 | 0.0139 | 0.0139 | 0.0139 | 0.0139 | 0.0138 | 0.0139 | 0.014 | 0.0139 |
| BJHR | 0.014 | 0.014 | 0.8475 | 0.0138 | 0.0138 | 0.0138 | 0.0138 | 0.0274 | 0.0139 | 0.0139 | 0.0139 |
| BJSY | 0.0159 | 0.0159 | 0.016 | 0.6836 | 0.0197 | 0.016 | 0.0199 | 0.016 | 0.1583 | 0.0195 | 0.0193 |
| LNDL | 0.0186 | 0.0187 | 0.0185 | 0.0186 | 0.6862 | 0.0186 | 0.0198 | 0.0186 | 0.1447 | 0.019 | 0.0186 |
| NXLW | 0.0145 | 0.0145 | 0.0146 | 0.0144 | 0.0144 | 0.855 | 0.0145 | 0.0145 | 0.0144 | 0.0145 | 0.0146 |
| NXPL | 0.014 | 0.014 | 0.0983 | 0.0144 | 0.0163 | 0.0907 | 0.6866 | 0.0145 | 0.0201 | 0.0154 | 0.0159 |
| NXZW | 0.0139 | 0.0139 | 0.014 | 0.0138 | 0.0139 | 0.0139 | 0.014 | 0.861 | 0.0139 | 0.0139 | 0.0138 |
| SDRZ | 0.0129 | 0.0129 | 0.0128 | 0.0128 | 0.0129 | 0.013 | 0.013 | 0.0128 | 0.871 | 0.013 | 0.0129 |
| SXYC | 0.0208 | 0.0208 | 0.0209 | 0.0208 | 0.0209 | 0.0209 | 0.0207 | 0.0208 | 0.125 | 0.6875 | 0.021 |
| SXYL | 0.0146 | 0.0146 | 0.0144 | 0.0148 | 0.0155 | 0.1753 | 0.0165 | 0.0146 | 0.0195 | 0.0153 | 0.6849 |

95% confidence intervals of M value of TRW:

| TRW | BJCY | BJHD | BJHR | BJSY | LNDL | NXLW | NXPL | NXZW | SDRZ | SXYC | SXYL |
| --- | --- | --- | --- | --- | --- | --- | --- | --- | --- | --- | --- |
| BJCY | 0.0195 | 0.0198 | 0.0196 | 0.0196 | 0.0196 | 0.0196 | 0.0195 | 0.0198 | 0.0392 | 0.0195 | 0.0195 |
| BJHD | 0.0134 | 0.0332 | 0.0182 | 0.0133 | 0.0134 | 0.0134 | 0.0133 | 0.0132 | 0.0133 | 0.0134 | 0.0133 |
| BJHR | 0.0134 | 0.0135 | 0.0335 | 0.0133 | 0.0133 | 0.0134 | 0.0133 | 0.0188 | 0.0134 | 0.0132 | 0.0134 |
| BJSY | 0.0152 | 0.0152 | 0.0153 | 0.0161 | 0.0180 | 0.0152 | 0.0181 | 0.0153 | 0.0354 | 0.0178 | 0.0176 |
| LNDL | 0.0176 | 0.0177 | 0.0175 | 0.0177 | 0.0187 | 0.0175 | 0.0188 | 0.0177 | 0.0387 | 0.0181 | 0.0176 |
| NXLW | 0.0139 | 0.0138 | 0.0139 | 0.0139 | 0.0139 | 0.0338 | 0.0138 | 0.0139 | 0.0138 | 0.0138 | 0.0140 |
| NXPL | 0.0134 | 0.0134 | 0.0397 | 0.0141 | 0.0166 | 0.0357 | 0.0207 | 0.0142 | 0.0210 | 0.0150 | 0.0156 |
| NXZW | 0.0134 | 0.0133 | 0.0133 | 0.0133 | 0.0134 | 0.0133 | 0.0134 | 0.0328 | 0.0133 | 0.0133 | 0.0132 |
| SDRZ | 0.0125 | 0.0125 | 0.0124 | 0.0124 | 0.0125 | 0.0126 | 0.0125 | 0.0123 | 0.0315 | 0.0126 | 0.0124 |
| SXYC | 0.0194 | 0.0195 | 0.0197 | 0.0197 | 0.0197 | 0.0195 | 0.0194 | 0.0195 | 0.0389 | 0.0196 | 0.0196 |
| SXYL | 0.0139 | 0.0139 | 0.0138 | 0.0142 | 0.0152 | 0.0366 | 0.0163 | 0.0139 | 0.0190 | 0.0149 | 0.0181 |

**Table S5** Results of variance partitioning showing the percentage of total explained variance (%explained) in SSR genotypes attributable to only climate, only population structure and joint effects of climate and population structure. Variance (inertia) values are given.

*Eucryptorrhynchus brandti*

| Effect | Variance | Explained (%) |
| --- | --- | --- |
| Total variance (Explained + unexplained) | 954.68 |  |
| Total explained variance (climate+population structure+climate/population structure) | 94.86 |  |
| Climate | 89.11 | 93.94 |
| Population structure | 28.19 | 29.71 |
| Joint climate/population structure | -22.44 | 0 |

*Eucryptorrhynchus scrobiculatus*

| Effect | Variance | Explained (%) |
| --- | --- | --- |
| Total variance (Explained + unexplained) | 2597.87 |  |
| Total explained variance (climate+population structure+climate/population structure) | 1707.56 |  |
| Climate | 690.49 | 40.44 |
| Population structure | 890.30 | 52.14 |
| Joint climate/population structure | 126.76 | 7.42 |

**
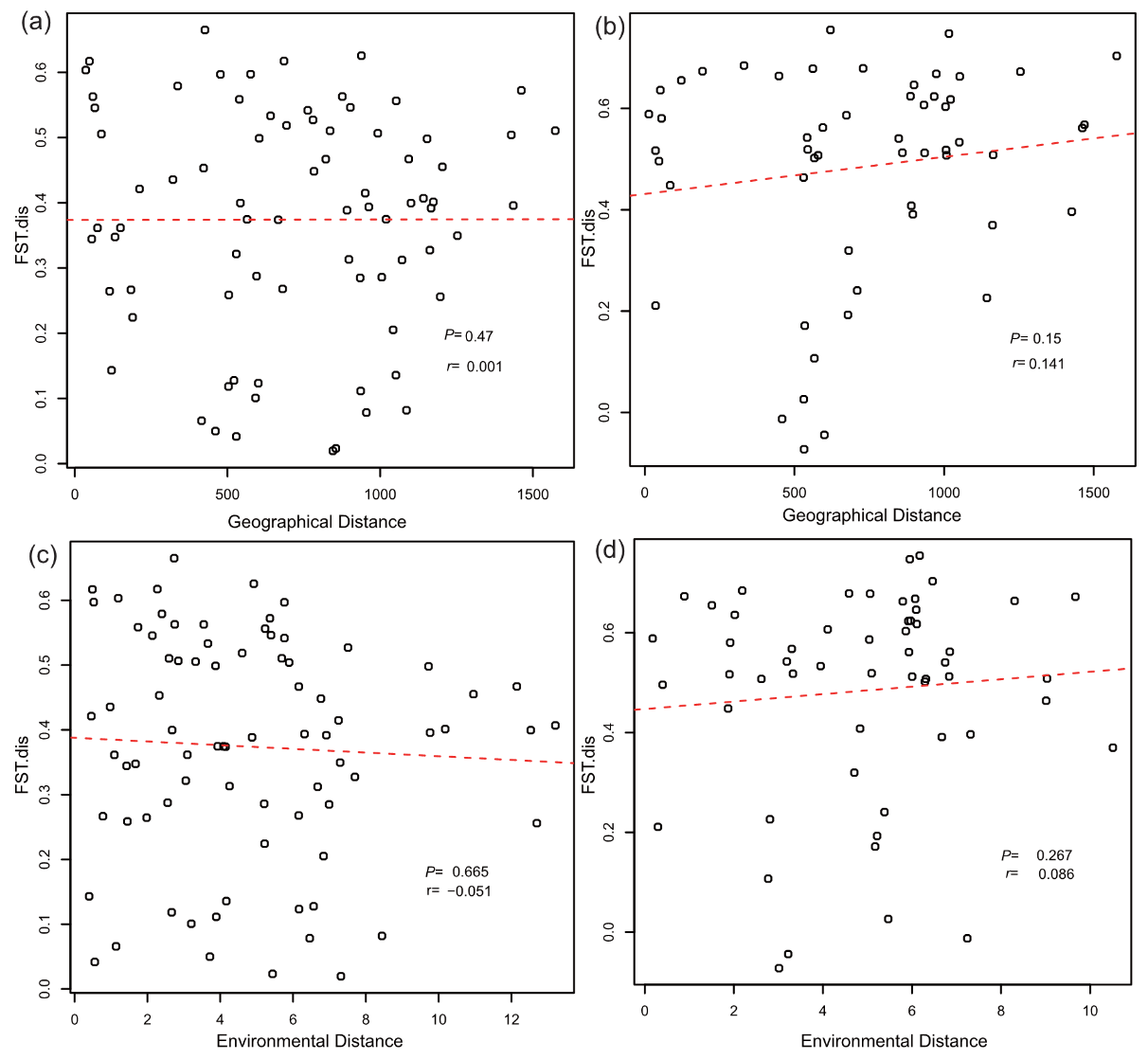
**

**Figure S1** Isolation by distance (IBD) (a, *Eucryptorrhynchus brandti*; b, *E. scrobiculatus*) and isolation by environment (IBE) (c, *Eucryptorrhynchus brandti*; d, *E. scrobiculatus*). And *r*, stands for slope; *p*, stands for significance.


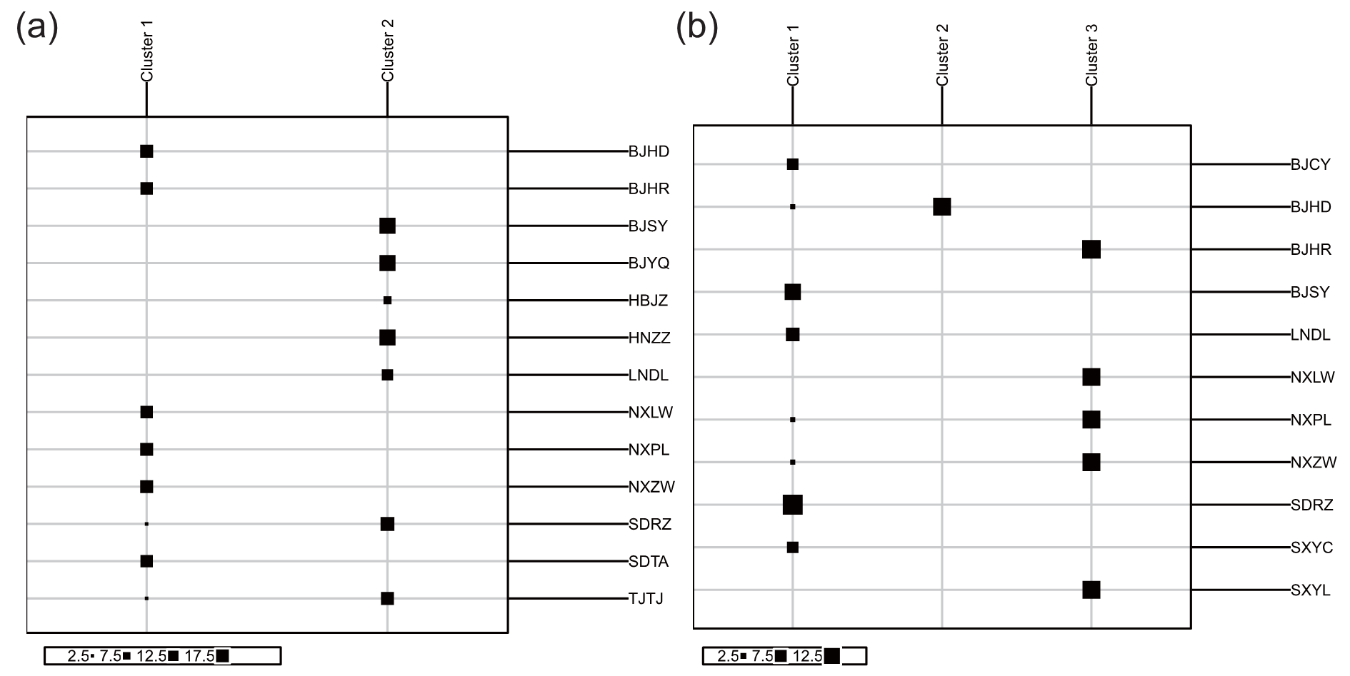


**Figure S2** Population genetic structure inferred from discriminant analysis of principal components (DAPC) (a, *Eucryptorrhynchus brandti*; b, *E. scrobiculatus*) analysis based on microsatellites.
